# Molecular Mechanisms of the Co-Evolution of Wheat and Rust Pathogens

**DOI:** 10.3390/plants12091809

**Published:** 2023-04-28

**Authors:** Emmanuel N. Annan, Li Huang

**Affiliations:** Department of Plant Sciences and Plant Pathology, Montana State University, Bozeman, MT 59717-3150, USA; gorbom1993@gmail.com

**Keywords:** wheat, rust, effector, NLR, resistance, co-evolution, sRNA

## Abstract

Wheat (*Triticum* spp.) is a cereal crop domesticated >8000 years ago and the second-most-consumed food crop nowadays. Ever since mankind has written records, cereal rust diseases have been a painful awareness in antiquity documented in the Old Testament (about 750 B.C.). The pathogen causing the wheat stem rust disease is among the first identified plant pathogens in the 1700s, suggesting that wheat and rust pathogens have co-existed for thousands of years. With advanced molecular technologies, wheat and rust genomes have been sequenced, and interactions between the host and the rust pathogens have been extensively studied at molecular levels. In this review, we summarized the research at the molecular level and organized the findings based on the pathogenesis steps of germination, penetration, haustorial formation, and colonization of the rusts to present the molecular mechanisms of the co-evolution of wheat and rust pathogens.

## 1. History of Wheat-Rust Co-Existence

Wheat refers to the cultivated *Triticum* spp., including the most common hexaploid bread wheat (*T. aestivum* L.) and the tetraploid durum wheat (*T. turgidum* var. *durum*). These cereal crops are the staple food in most developing countries and are the most traded grains globally. Wheat rust is among the earliest documented plant diseases, dating back to Aristotle’s time (384–322 B.C.) [1]. Epidemics of rust diseases were a reason for an ancient practice of Robigus, the rust god [2], and now the diseases are still one of the major constraints for wheat production worldwide [3]. There are three fungal species from the genus of *Puccinia*, *P. triticina* (*Pt*), *P. graminis* f. sp. *tritici* (*Pgt*), and *P. striiformis* f. sp. *tritici* (*Pst*) causing leaf, stem and stripe rust on wheat, respectively. The *Pgt* was first described with details by two Italian scientists, Fontana and Tozzetti, independently in 1767 and named by Persoon in 1797 [1]. Anton De Bary found barberry as an alternative host of *Pgt* in 1865 [1]. The three species of rusts are obligate biotrophic parasites that require living host cells to grow and reproduce [4]. However, the parasites can remain alive as spores in the absence of a living host for a period of time depending on the conditions [5]. Five types of spores are produced by the three rust pathogens, production of urediniospores, and teliospores on the primary grass hosts such as wheat by asexual reproduction. Teliospores germinate to produce basidiospores infect an alternative host to form pycniospores (spermatia) and produce aeciospores by sexual reproduction on the alternative host [6,7].

Wheat and rust have co-existed for >8000 years [8]. With the introduction of advanced molecular technologies, wheat and rust genomes have been sequenced, and interactions between the host and the rust pathogens have been extensively studied at molecular levels. In this review, we summarized the research at the molecular level and organized the findings based on the pathogenesis steps of germination, penetration, haustorial formation, and colonization of rusts to present the molecular mechanisms of the co-evolution of wheat–rust pathogens.

## 2. No Interactions during Rust Germination

Rust infections on their primary grass hosts, including wheat, start from aeciospores/urediniospores landing on the leaf surfaces. Under moist conditions, free water is required for spore germination, a process that occurs at night in the natural environment. Expression analysis using a cDNA library from germinated *Pst* urediniospores revealed germination stage-specific-expressed genes [9]. Over 60% of the genes were involved in primary metabolism (42.6%) and protein synthesis (21.6%). Some of the stage-specific-expressed *Pst* genes shared significant homology with known virulence factors such as HESP767 of flax rust and PMK1, GAS1, and GAS2 of rice blast fungus [9].

The germination of spores does not require living hosts, and the process can happen on a plastic surface when free water is present [9]. However, germination is inhibited by endogenous self-inhibitors released from the spores if the population density floated on the water is too high [10]. Each rust species has its optimum germination temperature in the range of 11~20 °C. Neither host genotypes (resistant or susceptible) nor host extracts [11] affect spore germination rates, thus suggesting that host defense responses did not happen at the stage of spore germination before penetration [12].

## 3. Molecular Interactions during Penetration and Haustoria Formation

**Figure 1:**
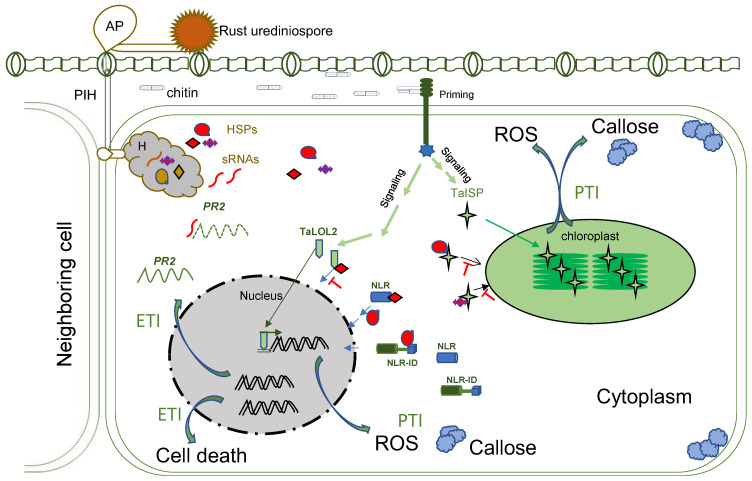
Molecular interactions with wheat host during rust infection. AP: appressorium; PIH: primary infection hyphae; H: haustorium; HSPs: haustorial secreted proteins; sRNA: small RNA; PR2: pathogenesis-related protein 2; ROS: Reactive Oxygen Species; TaISP: *Triticum aestivum* iron-sulfur protein; TaLOLs: *Triticum aestivum* LSD-One-Like 2; NLR: nucleotide-binding and leucine-rich repeat; NLR-ID: NLR–integrated domain; PTI: pathogenic-associated molecular pattern-triggered immunity; ETI: effector-triggered immunity.

After germination, the germ tubes grow perpendicular to leaf veins until they encounter stomata. The topology of specific host guard cells plays a vital role in stomata identification, known as thigmotropism. Formation of an appressorium is induced over a stoma around 4~16 h post-germination [13]. Then, an appressorium forms a penetration peg to initiate penetration, and a substomatal tube grows between the two guard cells of the host, a mechanism previously determined to be light-dependent [14]. Penetration is shortly followed by substomatal growth, including the formation of primary infection hyphae (PIH) that grow intracellularly until they encounter mesophyll cells. Once a PIH contacts a living mesophyll cell, the tip of the PIH differentiates to a haustorial mother cell (HMC), and a haustorium is induced (Figure 1). The HMC located outside of the mesophyll cell degrades a tiny hole on the cell wall and invaginate the plasma membrane to form an intracellular young haustorium [15] at about 24 h post-infection (hpi), and a mature haustorium as early as 48 hpi [16]. If the host cell in contact with the PIH is dying, the PIH differentiation stops, and hyphae will continue to grow to find another viable cell [16]. The mechanism to govern this process is still unknown.

During penetration of the host cell, a fungal cell wall component chitin can be an elicitor to trigger the wheat defense response [17], with the signatures of bursts of reactive oxygen species (ROS) such as H_2_O_2_ and increased callose deposition around the penetration sites [18,19], known as pathogenic-associated molecular pattern-triggered immunity (PTI). The host PTI is regulated by priming, signal cascades, and movements of transcription factors to the nuclear to activate more genes or movements of nucleus-encoded proteins to chloroplasts to generate ROS and callose. Wheat transcription factors TaLOL2, TaCBF1d, and a copper–zinc superoxide dismutase TaCZSOD2 are known to be positively involved in PTI [20,21,22].

In a successful rust infection, the pathogen must have overcome this layer of host defenses. Studies of rust whole-genome sequences revealed that each rust genome contains large annotated proteins with a secretory peptide, >600 in the *Pt* genome [23], >1000 in *Pgt* [24,25], and 700~1088 in *Pst* [26,27]. When wheat plants were challenged with mutants of several *Pst* secretory proteins, the plants showed large amounts of H_2_O_2_ accumulation and increased callose deposition around the penetration sites, suggesting those extracellularly secreted rust proteins (virulence effectors) could suppress PTI [18,19]. With the developments of a barley stripe mosaic virus (BSMV) mediated host-induced gene silencing (HIGS) (BSMV-HIGS) assay [28,29] and a bacterial type III secretion system (T3SS)-mediated fungal effector delivery assay for wheat [30], functions of many more rust secretory proteins have been revealed (Table 1).

At the early penetration stage, rusts deploy effectors to interfere with different steps of host PTI operated by the nucleus or organelles, such as chloroplasts (Table 1). For example, the *Pst* effector PstGSRE1, a glycine–serine-rich protein, disrupted the movement of TaLOL2 to the nucleus [21]. TaLOL2 is a transcription factor promoting ROS. PstGSRE4 targeted the wheat copper–zinc superoxide dismutase TaCZSOD2, an enzyme positively involved in PTI [20]. The PstPEC6 effector interacted with the wheat adenosine kinase to hamper ROS accumulation and callose deposition [31]. Genes encoding *PEC6* homologs are conserved among *Pst* isolates and highly similar among three rust species [28], suggesting a conserved strategy for suppressing PTI among the rusts. *Pst* effector PsSpg1 interacted with TaPslPK1 to promote its nuclear localization and subsequent phosphorylation of TaCBF1d and degradation. TaCBF1d is a crucial transcription factor in activating PTI [22]. Inactivation of TaPslPK1 rendered wheat with broad-spectrum resistance to rusts [22]. These studies also found that rust uses multiple effectors to target the same host protein. For example, more than five *Pst* effectors, including *Pst*_4 and *Pst*_5, interact with the wheat chloroplast protein TaISP (cytochrome *b*6-f complex iron-sulfur subunit) in the cytoplasm and prevent the protein from entering the chloroplasts (Table 1). TaISP accumulation is required for chloroplast-derived ROS production [32]. This functional redundancy gives the pathogen the ability to afford losing/mutating a few of its effectors.

**Table 1:** Characterized rust effectors.

Effector	Target	Function/Purpose	Reference
PstPEC6	TaADKs/nucleus	Hamper ROS accumulation and callose deposition	[31]
PstGSRE1	TaLOL2/nucleus	Stop TaLOL2 movement to nucleus/suppressing PTI	[21]
PsSRPKL	Unknown nucleus gene	Hamper ROS accumulation	[18]
PstGSRE4	TaCZSOD2/nucleus	Hamper ROS accumulation and callose deposition	[20]
Pst02549	TaEDC4/nucleus	mRNA decapping protein 4/manipulating host P-bodies	[33]
Pst03196	Chloroplast protein	Hamper ROS accumulation	[33]
Pst18220	Chloroplast protein	Hamper ROS accumulation	[33]
PstShr7	unknown	Suppressing PTI and HR	[34]
PstShr1~9	unknown	Suppressing HR	[34]
Pst_8713	Unknown nucleus gene	Suppressing PTI and HR	[35]
Pst_12806	TaISP/chloroplast	Block TaISP entering chloroplasts/Reduce chloroplast-derived ROS production	[36]
Pst_4	TaISP/chloroplast	Block TaISP entering chloroplasts/Reduce chloroplast-derived ROS production	[32]
Pst_5	TaISP/chloroplast	Block TaISP entering chloroplasts/Reduce chloroplast-derived ROS production	[32]
PsSpg1	TaPsIPK1/nucleus	Phosphorylation of TaCBF1d/Reduced ROS accumulation and callose deposition	[22]

Once the host PTI was suppressed, rust could form haustoria, and haustorial-specific/enriched genes could start to express or upregulate [37] as an effector repertoire. Transcriptomic studies of multiple isolates of each *Pst*, *Pgt*, and *Pt* revealed that the *Pst* had 1989 differential expressed genes in haustoria; 400 possess a secretion peptide, and >40% of the genes are involved in metabolic processes and translation [38], all six *Pt* races had 456 haustorial secreted proteins (HSPs) [39], and four different *Pgt* isolates had 520 HSPs [25]. About 77% of the *Pgt* haustorial secreted genes are heterozygous and polymorphic in the coding sequences, with a single nucleotide polymorphism (SNP) of 17.72/kb among isolates [25], suggesting large variations in HSPs among rust races. However, ~10% of the HSPs had no polymorphism, revealing highly conserved effectors. This pool of rust effectors facilitates further pathogen manipulation to suppress the host defense or hijack host cell machinery to establish artificial nutrient sinks that lead to pathogen feeding and profuse growth [15].

In addition to the deployment of effectors to attack crucial components of PTI/ effector-triggered immunity (ETI), rust produces small RNAs (sRNA) as important pathogenicity factors to impair host immunity [40]. For example, A 21-nt microRNA of *Pst (Pst-milR1)* was found to bind the wheat *PR2* gene to reduce the transcript abundance of the gene and suppress the host defense during infection. PR2 is a β-1, 3-glucanase with antifungal property [41]. High production of PR2 is the result of active ETI. Silencing the precursor of the *Pst-milR1* resulted in wheat resistance to the pathogen [40]. A transcriptomic study on wheat–*Pst* interaction at 7 dpi discovered differential expression profiles of sRNAs from wheat and *Pst* [42]. More 35-nt and less 24-nt sRNAs from the *Pst* infection wheat than the uninfected plant. *Pst* produced abundant sRNAs almost entirely of 19–23-nt in sizes [42]. It is believed that these sRNAs are used to regulate transcripts of both native and cross-species post-transcriptionally during the interaction [42]. Details on their targets and mode of actions are hot topics of current studies on wheat–rust interactions.

Accordingly, wheat has a sophisticated surveillance system to detect rust effectors and activates even stronger defense responses, known as ETI. For example, wheat Sr35 detects *Pgt* AvrSr35 and mediates resistance to stop the PIH growth before the formation of haustoria [43]. So far, there are 231 designated rust resistance genes [44,45,46,47,48,49], including 83 leaf rust resistance (*Lr*) genes, 64 stem rust resistance (*Sr*) genes and 84 stripe rust resistance (*Yr*) genes. So many rust resistance genes implicate a high variation from their counterpart.

The first cloned wheat rust resistance gene *Lr21* [50] shed light on the defense mechanism of wheat ETI against leaf rust. *Lr21* encodes a protein with nucleotide-binding and leucine-rich repeat (NLR) domains, mediating resistance with hypersensitive cell death and high pathogenesis-related protein (PR) productions. Up to now, 29 rust resistance genes have been cloned [51,52,53,54]. Twenty-three of the 29 R proteins (79%) belong to the NLR class (Table 2). After the bread wheat whole genome was sequenced, sequence annotation revealed that the wheat genome contains ~2151 NLR-like genes [55]. These genes are arranged in 547 gene clusters and located at the distal ends of the chromosomes, known as recombination hotspots [55,56]. Many clusters contain genes with >75% similarity, suggesting that the genes in the same cluster were generated by duplication. In addition, some of the clusters contain genes encoding only part of an NLR protein, e.g., five genes encoded a toll/interleukin-1 receptor (TIR) without LRR [55], suggesting that deletion mutations happened after gene duplications. These findings imply that the wheat genome has a rich number of diversified NLRs as an inventory ready to detect a large variety of effectors and generate more categories of NLRs through recombination.

The NLR-like proteins identified in the wheat genome are classified as the majority to be the classical NLRs containing an N-terminal coiled-coil (CC)/TIR + NB + LRR domains [54] and some NLR fusion proteins, e.g., an NLR fused with integrated domain (ID) homologous with proteins of different functions [54,57,58]. The abundance and locations of the wheat NLR proteins and their structures left footprints on how the new genes were evolved, and what the possible modes of their defense actions are.

Classical NLRs are known to recognize pathogen effectors, also called avirulence (Avr) proteins, directly or indirectly [59]. Studies on the molecular interactions of three pairs of wheat Sr proteins and the matching Avr proteins, Sr35/AvrSr35 [43], Sr50/AvrSr50 [60], and Sr27/AvrSr27 [52], demonstrated that mutations at the DNA or expression level of the matching effector genes were the mechanisms of generating new *Pgt* virulence isolates. For example, the *Sr35*-mediated resistance stops the development of PIH before the formation of haustoria [43]. A transposon-mediated insertion in AvrSr35 created Sr35-virulent *Pgt* isolates with increased expression in a susceptible wheat line [43]. A spontaneous mutant with DNA insertion and loss-of-heterozygosity at the *AvrSr50* locus by somatic exchange compromised the interaction of Sr50/AvrSr50 and abolished *Sr50*-mediated resistance [60]. The *AvrSr27* locus encodes two related secreted proteins. The pathogen used copy number variation, deletion, and expression level polymorphism to compromise the Sr27 recognition [52]. To cope with the variations in effectors for escaping recognition, the wheat host also generated new resistance genes to recognize the new effector variants. For example, two pseudo-NLR-like genes in the D-genome recombined intragenically to produce a chimeric allele (*Lr21*) with new resistance specificity. The origin of *Lr21* in nature and the experimental reconstitution of the *Lr21* gene in wheat through intragenic recombinations suggested that wheat reuses the mutations accumulated in the NLRs to generate new disease specificity through intra- and/or inter-genic recombinations [61].

**Table 2:** Wheat rust resistance genes cloned.

Gene	R Protein Structure	Reference
*Lr1*	NLR	[62]
*Lr10*	NLR	[63]
*Lr13*	NLR	[64]
*Lr21*	NLR	[50]
*Lr22 a*	NLR	[65]
*Lr34/Yr18/Sr57/Pm38*	ABC transporter	[66]
*Lr42*	NLR	[67]
*Lr67/Yr46/Sr55/Pm46*	Hexose transporter	[68]
*Sr13*	NLR	[69]
*Sr21*	NLR	[70]
*Sr22 a*	NLR	[71]
*Sr22 b*	NLR	[51]
*Sr26*	NLR	[72]
*Sr27*	NLR	[52]
*Sr33*	NLR	[73]
*Sr35*	NLR	[74]
*Sr45*	NLR	[71]
*Sr46*	NLR	[75]
*Sr50*	NLR	[76]
*Sr60*	Tandem kinase	[77]
*Sr61*	NLR	[72]
*Sr62*	Tandem kinase	[53]
*SrTA1662*	NLR	[75]
*Yr5 a*	NLR	[78]
*Yr5 b*	NLR	[78]
*Yr7*	NLR	[78]
*Yr15*	Tandem kinase-pseudokinase	[79]
*Yr36*	Kinase-START	[80]
*YrAS2388*	NLR	[81]

There are 28 different types of IDs that have been identified to be present in either the *N*- or *C*-terminus of an NLR in wheat [55]. These IDs include kinase and DNA-binding domains, which function as signal transduction. An ID of a kinase or a DNA-binding in an NLR may help the NLR to initiate defense signaling through phosphorylation of transcription factors by the biochemical activity of kinase [82] or move directly to the nucleus with DNA-binding to the promoters of genes involved in defense responses. However, an ID such as NPR1 fused with an NLR protein is likely to serve as a molecular bait or decoy. NPR1 is a key transcriptional regulator in plant defense [83,84,85,86,87]. Wheat has 6 members of *NPR1*-like genes located in homoeologous groups 3 and 7. The group 3 *NPR1* genes regulate the salicylic acid (SA)-signaling pathway and the crosstalk between SA- and Jasmonic acid (JA)- pathways. The *Ta7ANPR1* locus in wheat encodes two types of NLRs, NB-ARC, and NB-ARC + NPR1, through alternative splicing [58]. Together with a CC + NB-ARC gene at a head-to-head orientation, it monitors the integrity of NPR1 proteins. Mutations in the Ta7ANPR1 activated resistance to rusts [58]. This finding suggests that some rust effectors attack wheat NPR1 to disrupt ETI. The variations in NLRs and diversified IDs of NLR-IDs suggest that wheat surveillance systems can detect rust effectors or monitor the cellular integrity of the host cells.

Three rust resistance genes, *Sr60*, *Sr62* and *Yr15*, encode proteins of tandem kinase (Table 2). The molecular basis of tandem-kinase-mediated resistance could be similar to the bacterial speck disease-resistant protein Pto. The tomato *Pto* gene encodes a serine-threonine protein kinase [88]. Two *Pseudomonas* effectors physically interact with the Pto kinase directly, and degradation/phosphorylation of the Pto kinase protein post-interaction is detected by Prf, an NLR protein. Defense response is activated by the Prf protein, and Pto serves as a decoy for Prf to monitor the integrity of its important kinase proteins [88].

Besides the tactics of escaping the host surveillance detection and suppressing the defenses of PTI/ETI, rust also plays a game to manipulate the host defense signaling. A study on the wheat–rust interaction by Nyamesorto et al. found that the *MYC4-1B* gene of wheat was highly upregulated in a susceptible line post-*Pgt* inoculation. MYC4 is a transcription factor positively involved in JA and JA-isoleucine accumulations [89]. There are two potential outcomes that benefit *Pgt* with high JA levels—the stomata opened up and the SA-signaling pathway suppressed. The SA-mediated pathway is known to be an effective defense response against biotrophic pathogens [90], including rusts. An opened-up stoma is required for rust appressoria to penetrate the host. Knockout of the *TaMYC4-1B* increased SA-mediated *PR* gene expressions and rendered new resistance to three rusts [91].

## 4. Molecular Interactions during Colonization and Nutrient Acquisition

After all the battles with wheat, ultimately, rust pathogens want to obtain nutrients from the host to grow and reproduce. Haustoria are believed to be the powerhouse of feeding sites for pathogen colony growth. The biotrophic nature and lifestyle of the rusts imply that the pathogen needs to manipulate the host cellular machinery to redirect the sink sources to haustorial sites. Understanding of the molecular interactions during this step is still limited. A wheat gene *Lr34* encoding an ABC transporter confers durable and non-specific resistance against biotrophic fungal pathogens [66]. *Lr34res* and *Lr34sus* differ by two nucleotides, and as a result, the LR34sus protein was undetectable in planta due to post-transcriptional regulation [92]. The substrate of the LR34 ABC transporter is phytohormone abscisic acid (ABA), the redistribution of ABA by LR34 has been found to have a major effect on the transcriptional response and physiology of the host, resulting in resistance [92]. In another study, a mutation on the hexose transporter gene *Lr67* [68] was found to confer non-specific resistance against four biotrophic fungal pathogens of three rusts and powdery mildew. LR67sus transporter has a high affinity to glucose. Two amino acids different in the LR67res protein reduced glucose uptake, of which reduced the growth of multiple biotrophs [68]. The discovery showed us a passive non-battle strategy for the host to combat biotrophs. The findings also suggested conserved molecular mechanisms among biotrophic pathogens for colonizing and acquiring nutrients from the wheat host.

## 5. Closing Remarks

**Figure 2:**
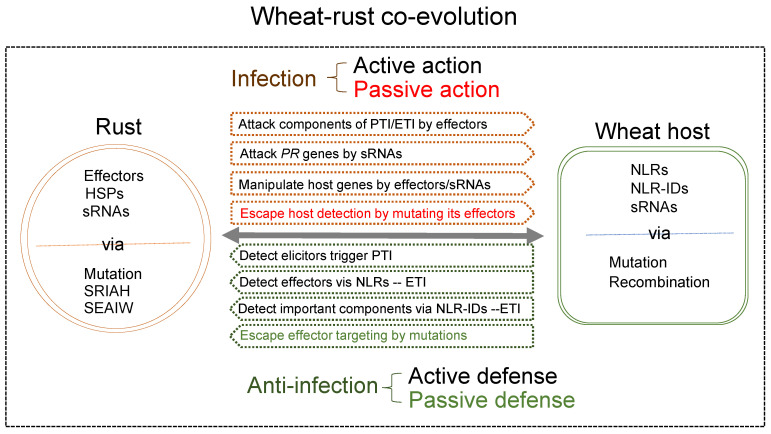
Summary of the mechanisms during wheat–rust co-evolution. HSPs: haustorial secreted proteins; sRNA: small RNA; SRIAH: sexual recombination in alternative hosts; SEAIW: somatic exchange asexually in wheat; NLR: nucleotide-binding and leucine-rich repeat; PTI: pathogenic-associated molecular pattern-triggered immunity; ETI: effector-triggered immunity.

From the current not-yet-comprehensive studies at the molecular levels of wheat, rusts, and their interactions, we can partially restore or speculate the events that happened in nature during the races between wheat and rust over thousands of years of co-evolution (Figure 1 and Figure 2). Based on the functions of cloned resistance genes, the most common strategy of wheat against rusts is the active defense upon recognition. Passive defense is an alternative, requiring changes in the host genes that could be deleterious to the host. Rusts mutate their effector genes at the DNA level, expression level or copy numbers to escape detection or deliver effectors/sRNAs to sabotage host defense signaling or manipulate host cellular machinery. An arms race between the effectors and R proteins is the central theme during the co-evolution. Monoculture using a single race-specific *R* gene could intensify the race due to the high selection pressure on the pathogen populations. As witnessed within the Great Plains of the United States, the highly resistant cultivars became susceptible after several years of production, when wheat cultivars with a single new rust resistance gene were grown over a large area; this is known as the “boom and bust” cycle of the cereal rust resistance genes [93]. The strategies of the infection and anti-infection of rust pathogens and wheat host, their genome compositions and organizations suggested that both sides of the organisms have the ability, and are always prepared, to adapt to changes for the opposite party. The long history of their co-existence even after human’s intervention via resistance breeding suggests that rust pathogens will unlikely become extinct. Checks and balances seem to be the means for maintaining cosmic diversity. If completely wiping out something is impossible, then finding a state of peaceful co-existence should be an alternative. The current switch of using genes with partial resistance instead of complete resistance for rust resistance breeding reflects the idea for peaceful co-existence. A complete understanding of the molecular mechanisms of wheat–rust interactions will provide the knowledge base for searching and establishing the points of balance and maintaining the peaceful state for wheat and rust pathogens. Resistance genes that recognize crucial and possibly conserved effectors to the pathogens should have long-lasting effectiveness because mutations in those effectors will significantly reduce the pathogen fitness. However, strategical deployment of the *R* genes will be more critical in maintaining the balance. Pyramiding multiple *R* genes will enhance wheat resistance, but meanwhile it will impost selection on the pathogens. The level of resistance or the number of *R* genes to stack will translate to the level of selection pressure for the pathogens. High selection pressure will result in purified selection for a super-virulent race, leading to a bust of the resistant variety. Alternatively, growing mixtures of varieties each with partial resistance conferred by different *R* genes in the same field will have low selection pressure on the pathogen, and thus, will maintain the population diversity and balance between the host and pathogen.

## Data Availability

Not applicable.
